# On multifactorial drivers for malaria rebound in Brazil: a spatio-temporal analysis

**DOI:** 10.1186/s12936-021-04037-x

**Published:** 2022-02-17

**Authors:** Mario J. C. Ayala, Leonardo S. Bastos, Daniel A. M. Villela

**Affiliations:** Fundação Oswaldo Cruz (Fiocruz), Programa de Computação Científica, Av. Brasil, 4365—Manguinhos, Rio de Janeiro, 21040-900 Brazil

**Keywords:** *Plasmodium vivax*, *Plasmodium falciparum*, Malaria rebound, Spatio-temporal Bayesian model

## Abstract

**Background:**

Malaria incidence in Brazil reversed its decreasing trend when cases from recent years, as recent as 2015, exhibited an increase in the Brazilian Amazon basin, the area with the highest transmission of *Plasmodium vivax* and *Plasmodium falciparum*. In fact, an increase of more than 20% in the years 2016 and 2017 revealed possible vulnerabilities in the national malaria-control programme.

**Methods:**

Factors potentially associated with this reversal, including migration, economic activities, and deforestation, were studied. Past incidences of malaria cases due to *P. vivax* and *P. falciparum* were analysed with a spatio-temporal Bayesian model using more than 5 million individual records of malaria cases from January of 2003 to December of 2018 in the Brazilian Amazon to establish the municipalities with unexpected increases in cases.

**Results:**

*Plasmodium vivax* incidence surpassed the past trends in Amazonas (AM), Amapá (AP), Acre (AC), Pará (PA), Roraima (RR), and Rondônia (RO), implying a rebound of these states between 2015 and 2018. On the other hand, *P. falciparum* also surpassed the past trends in AM, AC, AP, and RR with less severity than *P. vivax* incidence. Outdoor activities, agricultural activities, accumulated deforestation, and travelling might explain the rebound in malaria cases in RR, AM, PA, and RO, mainly in *P. vivax* cases. These variables, however, did not explain the rebound of either *P. vivax* and *P. falciparum* cases in AC and AP states or *P. falciparum* cases in RR and RO states.

**Conclusion:**

The Amazon basin has experienced an unexpected increase in malaria cases, mainly in *P. vivax* cases, in some regions of the states of Amazonas, Acre, Pará, Amapá, Roraima, and Rondônia from 2015 to 2018 and agricultural activities, outdoor activities, travelling activities, and accumulated deforestation appear linked to this rebound of cases in particular regions with different impact. This shows the multifactorial effects and the heterogeneity of the Amazon basin, boosting the necessity of focusing the malaria control programme on particular social, economic, and environmental conditions.

**Supplementary Information:**

The online version contains supplementary material available at 10.1186/s12936-021-04037-x.

## Background

Control of malaria transmission depends on multiple factors involving vector control, treatment of infected individuals, and prophylactic actions, requiring permanent attention and control of exogenous conditions. In the case of Brazil, the country reduced its malaria incidence due to both *Plasmodium vivax* and *Plasmodium falciparum* until the year of 2015 but data from recent years exhibited an increase in notified cases [1]. In fact, an increase of 25% between 2016 and 2017 revealed the vulnerability of these efforts [2, 3].

The Amazon basin has an area greater than many countries and a vast species diversity due to the Amazon Forest and preserving natural resources. These regions have accounted for 99.5% of cases registered in Brazil in the last years [4]. Urban developments, deforestation activities, and different economic activities, such as mining, pose challenges to a large malaria control programme.

Previous studies established a set of plausible factors related to economic activities [5–8]. However, the unexpected increase of cases from 2016 to 2018 in the Brazilian Amazon region was not evaluated. In addition, the Amazon region presents divergences in malaria incidence between municipalities and States [9], and some of the previous studies only focused on specific regions.

Here, incidence trends from 2003 to 2015 were analysed, defining an expected trend using a statistical model. This model permits to predict incidences in the following years according to the trends. The observed incidences in all municipalities in the Amazon region from 2016 to 2018 were compared to the expected values via analyses using a Bayesian model to investigate possible relationships between this increase and multiple factors.

## Methods

### Epidemiological data

A dataset of 5,972,715 individual records of malaria cases from January of 2003 to December of 2018 was obtained, with epidemiological data from the States of Amazonas (AM), Acre (AC), Pará (PA), Amapá (AP), Roraima (RR), Rondônia (RO), Mato Grosso (MT), Maranhão (MA) and Tocantins (TO) (see study area in additional file 1). Each record included: notification date, infection data, notification type (active or passive), notification location, occupation, infection location, examination results, periodic control. These data were provided by the Brazilian Ministry of Health through a request for non-identified data from the Brazilian Information System of Epidemiological Surveillance (SIVEP) covering the years of study. These data were later available on an integrated dataset provided by Baroni et al. [10]. The study included only notifications of cases by *P. vivax* and *P. falciparum*. Exclusion criteria were: periodic-control records after treatment (1,041,397 records); inconsistent information in dates (10,436 records); notification without complete information (957,879 records). Other malaria species and mixed malaria cases (44,920 records). Thus, a total of 3,918,083 individual records were analysed, which included 3,232,766 observations of cases by *P. vivax* infection and 685,317 observations of *P. falciparum* infections.

### Population data and Brazilian maps

Annual records of population sizes per municipalities in the Amazon basin from 2003 to 2018 were obtained from the Brazilian Institute of Geography and Statistics (IBGE) [11]. Brazilian maps were also acquired from the maps and cartography website of IBGE [12].

### Deforestation data

The accumulated annual amount of deforestation in km^2^ per municipality was obtained from the PRODES project of the National Institute of Spatial Research [13].

### Bayesian models and incidence prediction

A Bayesian model was used to analyse the malaria incidences of 808 municipalities in 192 months (from January of 2003 to December of 2015). Spatial variation was labelled as *i*  = 1, …, 808 and temporal variation was labelled as *t*  = 1, …, 192. The number of cases *y*_*it*_ in municipality *i* at month *t* was modelled as counts using a Poisson distribution with mean *λ*_*it*_:

1 $$y_{it} \sim Poisson\left( {\lambda_{it} } \right),$$

where *λ*_*it*_  =  *ρ*_*it*_*ϵ*_*it*_ with *ρ*_*it*_ as incidence rate and *ϵ*_*it*_ as offset; the sizes of municipality populations per 100,000 inhabitants are applied in the model as offset terms. Here, incidence rate is described by a linear predictor in logarithmic scale:

2 $$\eta_{it} = \log \left( {\rho_{it} } \right) = \alpha + \gamma_{k} + \phi_{l} + \beta_{i} ,$$

with *α* as average incidence in all municipalities, *γ*_*k*_ as month effect (*k*  = 1, …, 12) according with Random Walk Model of order two (rw2) [14], *ϕ*_*l*_ as year effect (*k* = 1, …, 16) according with independent Gaussian random effects (iid) and *β*_*i*_ as municipality effect according with “iid”. In a second representation a spatial and temporal effect is added:

3 $$\eta_{it} = \log \left( {\rho_{it} } \right) = \alpha + \gamma_{k} + \phi_{l} + \beta_{i} + \nu_{i} + \delta_{t} ,$$

where *ν*_*i*_ is a spatial random effect according to Besag–York–Mollie (bym) specification [15], and *δ*_*t*_ as temporal random effect derived from the Random Walk Model of order one (rw1) [14]. Estimation with these models were obtained with implementations using the Integrated Nested Laplace Approximation (INLA) [16, 17]. This approach is a Bayesian method that provides computational efficiency through INLA package in R. The default prior distributions in INLA [logGamma(0,0.00005)] were used on the log of unstructured precision in iid, rw1 and rw2 random effects. The default prior distribution [logGamma(0,0.005)] were used on the log of unstructured precision and on the log of spatial precision in bym random effect.

Two models were used: a base model (model 1 Eq. 2) and base model plus spatial and temporal effects (model 2 Eq. 3). Data on malaria cases from 2003 to 2015 were analysed applying these models per state using INLA and evaluated using the deviance information criterion (DIC) that assesses the quality of these Bayesian models [18]. Model 2 was chosen for predicting the incidences from 2016 to 2018, after resulting in the least DIC for all states (see additional file 2).

### Difference between predictions and observed incidences

Incidence data from January 2003 to December 2015 were analysed for modelling and fitting, which permitted to obtain predictions from 2016 to 2018. The differences between the observed incidences and predicted incidences were calculated per month in each municipality from January 2016 to December 2018. In order to find the impact by year, the average differences between predictions and observed incidences were calculated per year (2016, 2017 and 2018). These yearly average differences were used to map the impact by year, implying an estimation discrepancy.

Difference maps displayed the average difference of prediction in 2016, 2017 and 2018 for *P. vivax* and *P. falciparum* incidence. These maps were generated in R using lattice package [19].

A positive difference is defined as the incidence above prediction, representing an unexpected increase of cases given past trends. Municipalities with a positive difference above 25 cases of *P. vivax* and above 10 cases of *P. falciparum* were selected for illustrating heat maps. Such maps were generated with these estimations using R using ggplot2 library [20].

### Model with deforestation, occupation categories, and imported cases

Incidence data also reported the individual occupation and place of infection (SIVEP data). Occupation categories are agriculture, livestock, housing, tourist, gold-mining, vegetable extraction, hunting/fishing, road building, mining, traveler, and other ones. The proportions of occupations per municipality was obtained after grouping occupation categories: agricultural activities (agriculture and livestock), outdoor activities (vegetable extraction, hunting/fishing and road building), mining (gold mining and mining), travelling and housing. In addition, the annual proportion of imported cases was calculated with malaria cases with countries in the border as likely place of infection to evaluate the impact of imported cases in last three years. PRODES reported data on the accumulated deforestation by municipality per year. Due to this reason, all variables in this model with covariates were yearly counts. The annual incidence was analysed using as covariates the annual accumulated deforestation in km^2^, the annual proportion of cases by occupation categories, and imported cases by municipality from 2003 to 2018.

The Bayesian model represents malaria incidence of 808 municipalities in 16 years (from 2003 to 2018) adding a random effect of deforestation and each occupation category. Spatial variation was labelled as *i*  = 1, …, 808 and temporal variation was labelled as *t*  = 1, …, 16. *y*_*it*_ is malaria incidence in municipality *i* at year *t*. Variable *y*_*it*_ models counts described by a Poisson distribution with mean *λ*_*it*_ (see Eq. 1). Equation 4 is derived on the structure of Eq. 3 adding the variable effect before 2016 and after 2015:

4 $$\eta_{it} = \log (\rho_{it} ) = \alpha + \gamma_{k} + \phi_{l} + \beta_{i} + \nu_{i} + \delta_{t} + \sum\limits_{n = 1}^{7} {(\zeta_{1,\,n} x_{n,\;i} + \zeta_{2} \omega_{i} + \zeta_{3,\,n} \omega_{i} x_{n,\;i} )} ,$$

*x*_*n,i*_ represents the variable *n* at year *i*, *ω*_*i*_ is a binary variable for representing the period from 2016 to 2018 (*ω*_14_, *ω*_15_, *ω*_16_  = 1 and *ω*_*i*_  = 0 for *i*  < 14), *ζ*_1, *n*_ is the random effect of variable *n* in all period, *ζ*_2_ is the random effect after 2015 and *ζ*_3, *n*_ is the random effect of variable *n* after 2015. Variables with unexpected increase in their effect on incidence after 2015, and variables with *ζ*_3, *n*_ intervals greater than 0 were selected for results.

Data preparation and model scripts are provided in the URL: https://github.com/Mario-Canon-Ayala/On-multifactorial-drivers-for-malaria-rebound-in-Brazil.

## Results

### Model validation and expected incidence after 2015

**Fig. 1:**
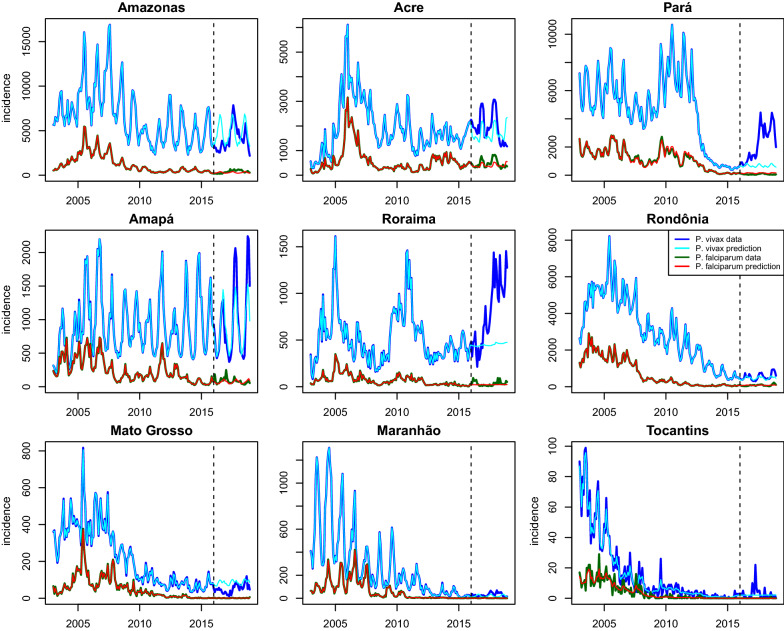
Comparison between model prediction and malaria incidence per state. Blue and cyan lines represent the observed incidences and predicted incidences for *P.* *vivax*; green and red lines represent observed incidence and predicted incidence for *P.* *falciparum*; figures illustrate incidence and prediction per month. Dashed lines indicated the moment (January 2016) when the prediction starts based on previous data

Analysis from statistical model permitted to generate samples that describe the time series of malaria incidences due to *P. vivax* and *P. falciparum* across cities and states in the Brazilian Amazon basin. These series can be compared to the observed incidence in all states from 2003 to 2015 for both *P. vivax* and *P. falciparum*, after fitting these models with the complete data from this period. The analysis is carried at municipality levels and presents the aggregate values at state levels. The predictions with the Bayesian model indicated trends with malaria incidence from 2016 to 2018 according to state-past trends (2003–2015), and the observed incidences were above predictions in some states (Fig. 1). This is the case of *P. vivax* incidence in Amazonas (AM), Acre (AC), Pará (PA), Amapá (AP), Roraima (RR), and Rondônia (RO) where observed incidence surpassed predictions indicating that these States experienced an unexpected growth of cases. This effect also occurred with *P. falciparum* incidence in AM, AC, AP, and RR with less severity than *P. vivax* incidence.

**Fig. 2:**
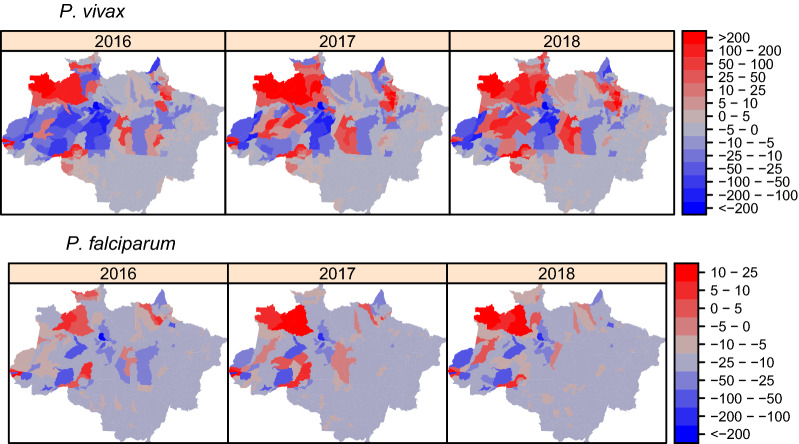
Monthly difference of prediction from 2016 to 2018 (average per each year). Red regions represent municipalities with a positive difference (incidence above prediction), and blue regions represent municipalities with a negative difference (incidence below prediction). The first row displays *P.* *vivax* prediction differences and the second row displays *P.* *falciparum* prediction differences

A set of municipalities presented incidence above predictions—positive differences—where the northwest region of Amazonas (AM), the northwest region of Acre (AC), and the northwest region of Rondônia (RO) obtained the highest positive differences for both *P. vivax* and *P. falciparum* incidence (Fig. 2). Positive difference for *P. vivax* also involved more regions than *P. falciparum* and five regions contained municipalities with positive difference above 50 monthly cases above prediction: northwest and centre region of Amazonas (AM) next to centre and south region of Roraima (RR), the northeast region of Pará (PA) with border area next to Amapá (AP) (Marajó region), southeast of Pará (PA), the northwest region of Acre (AC) and northwest region of Rondônia (RO) (Madeira-Mamoré region) next to the south of Amazonas (Purus region).

**Fig. 3:**
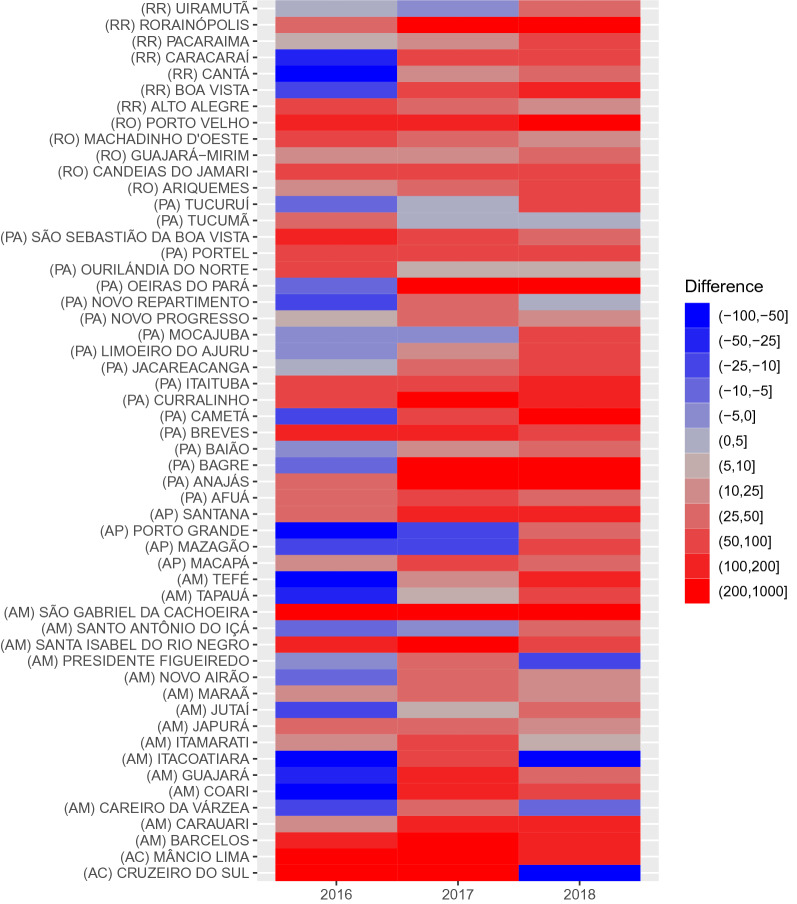
Municipalities with positive differences of prediction (average per each year) above 25 cases for P. vivax. The states of Roraima (RR), Rondônia (RO), Pará (PA), Amapá (AP), Amazonas (AM), and Acre (AC) contain these municipalities

A general rise in the number of municipalities with a positive difference of *P. vivax* occurred from 2016 to 2018 (see Fig. 3). The northwest region of AM started with positive differences, above 50, in 2016. The positive differences extended to all municipalities in the north region, southwest and south-center of AM, and most of the municipalities in RR between 2017 and 2018. The northwest region of RO maintained a similar pattern in 4 municipalities, and the positive difference in AM extended next to this region in 2018. The positive difference of the northeast region in PA and AP extended from 10 municipalities in 2016 (8 in PA and 2 in AP) to 17 municipalities in 2018 (13 in PA and 4 in AP). An increase also occurred in the southeast of PA, where positive difference extended from 2 municipalities in 2016 (2 in PA) to 4 municipalities in 2018 (3 in PA and 1 in AM). AC only maintained positive differences in two municipalities (Mâncio Lima and Cruzeiro do Sul).

**Fig. 4:**
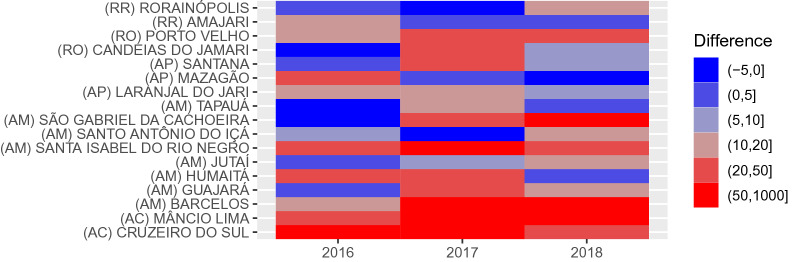
Municipalities with positive monthly differences of prediction (average per each year) above 10 cases of P. falciparum. The states of Roraima (RR), Rondônia (RO), Amapá (AP), Amazonas (AM), and Acre (AC) contain these municipalities

For *P. falciparum*, municipalities with positive difference increased in the northwest region of AM in São Gabriel da Cachoeira, Santa Isabel do Rio Negro, and Barcelos (see Fig. 4). In general, AM showed an increase in positive difference from 2016 to 2018, whereas the prediction difference decreased over time for AP state. The states RO and AC maintained positive differences in Porto Velho (RO), Mâncio Lima (AC), and Cruzeiro do Sul (AC).

### Model with deforestation, occupation categories, and imported cases

The analysis with the models by state estimated the random effect of each variable after 2015 (*ζ*_3, *n*_). Credibility intervals were entirely above 0, i.e., with a positive credibility interval, and mostly for *P. vivax* for a few variables. In particular, deforestation, outdoor activities, agricultural activities, and travelling activities appear linked to the unexpected increase of malaria cases in some states, mostly in *P. vivax* cases (see Table 1). Deforestation only obtained a positive credibility interval in *P. vivax* incidence in Roraima (RR) suggesting an effect due to deforestation in the unexpected increase in *P. vivax* incidence in this state after 2015. Outdoor activities (hunting/fishing, vegetable extraction, and road building) obtained an entirely positive credibility interval, also suggesting factors in the unexpected increase in malaria incidence in Amazonas (AM), either *P. vivax* and *P. falciparum* incidence, and *P. vivax* incidence in Pará (PA) after 2015. Agricultural activities might explain the unexpected increase in *P. vivax* incidence in Pará (PA) and Rondônia (RO), after 2015. Finally, the results suggested that travelling activities might explain the unexpected increase in *P. vivax* incidence in Rondônia (RO). A negative random effect in Rondônia (RO) was found only for a proportion of imported cases, implying that an increase in imported cases in this state represented a decrease in *P. vivax* incidence after 2015.

**Table 1:** Effects of variables after 2015 (*ζ*_3, *n*_)

State	Variable	Species	Mean (95% CI)	Unit
RR	Deforestation	*P. vivax*	3.53% (0.53–3.53)	km^2^
AM	Outdoor	*P. vivax*	8.25% (2.31–14.54)	1%
AM	Outdoor	*P. falciparum*	11.69% (0.64–23.98)	1%
PA	Agricultural	*P. vivax*	1.34% (0.01–2.69)	1%
PA	Outdoor	*P. vivax*	3.82% (0.46–7.28)	1%
RO	Agricultural	*P. vivax*	1.36% (0.48–2.26)	1%
RO	Travelling	*P. vivax*	21.27% (5.17–39.83)	1%
RO	Imported	*P. vivax*	− 7.61% (− 13.13 to − 1.74)	1%

The mean represents the effect (increase or decrease) on incidence with each variable increase. Mean in occupations (agricultural, outdoor, mining, and traveling) represents the increase in incidence for 1% more cases reported in each category

No variable was found significant to explain the trends in the random effect of either *P. vivax* and *P. falciparum* incidence in Acre (AC) or Amapá (AP).

## Discussion

*Plasmodium vivax* incidence surpassed the past trends in Amazonas (AM), Amapá (AP), Acre (AC), Pará (PA), Roraima (RR), and Rondônia (RO), implying a rebound of these states between 2015 and 2018. In addition, *P. falciparum* also surpassed the past trends in AM, AC, AP, and RR with less severity than *P. vivax* incidence. Outdoor activities, agricultural activities, accumulated deforestation, and traveling might explain the rebound in malaria cases in RR, AM, PA, and RO, mainly in *P. vivax* cases. None of the study variables explained the rebound of either *P. vivax* and *P. falciparum* cases in AC and AP, or the rebound of *P. falciparum* cases in RR and RO.

According to Fig. 2, some regions clusters concentrated the municipalities with incidences above predicted values in Amazonas, Acre, Roraima, Amapá, and Rondônia. Carlos et al. found the highest annual parasite index in these regions in the period from 2015 to 2016, and they also found that imported cases between regions and neighbouring countries played a role in malaria transmission [21]. Lana et al. also found some clusters in those regions that have provoked the majority of malaria cases in the Amazon basin from 2004 to 2018, similar to the current findings [22].

Results from the Bayesian models showed multiple factors playing a role in the rebound of malaria cases, especially the increase of *P. vivax* cases. The most important factors were related to human labour activities, such as outdoor activities (vegetable extraction, hunting/fishing, and road building), and agricultural activities might explain the rebound in *P. vivax* in Pará. In fact, the state of Pará has presented infrastructure projects that increase the malaria risk in this state [23], and this state has presented shortcomings in health access [24]. In addition, a previous study identified the land use for cattle production in the Carajás region of Pará as a risk factor between 2014 and 2018, although the current work found the principal unexpected contributor of cases in the Northwest region in Marajó region [25]. This study also identified mining as a risk factor in this region as a previous study found in Tapajós region [26]. However, the current calculated random effect did not show an unexpected increase in malaria risk by mining activities after 2015, suggesting that mining might maintain the same effect from previous years. Building, farming, and cattle production have represented a risk factor of malaria in this state, and results here suggest that they might have increased their impact between 2016 and 2018 [27].

Also, results here show an effect due to outdoor activities in the rebound of either *P. vivax* and *P. falciparum* cases in Amazonas state after 2015. This was the only state where the Bayesian analysis found a positive random effect, analysing *P. falciparum* cases after 2015, in agreement with the most affected region by *P. falciparum* cases located between Amazonas and Acre in previous works [21]. This state presented the most proportion of the indigenous population in Brazil, and the combination of hunting and fishing is the principal economic activity of this population. Results here suggest that these economic activities might have a factor in the increased malaria incidence after 2015 in this state. In fact, Martins et al*.* found a high risk of malaria cases in the indigenous population at this state between 2007 and 2019 [28]. Economic activities such as agriculture, mining, tourist, and vegetable—products extraction have raised malaria transmission in the northwest of Brazil. However, the results here suggest that these activities maintained the same effect over malaria incidence in Amazonas between 2015 and 2018 in comparison to the previous years [5].

Results from applying the Bayesian model showed that agricultural activities and travelling activities might explain the rebound of *P. vivax* cases in Rondônia after 2015. This state has developed infrastructure projects and economic activities increasing malaria risk since before 2000, but it has experienced a decrease in malaria cases in the last decade [29]. This state has also presented high deforestation rates related to agricultural activities suggesting that this economic activity might have increased malaria cases from 2015 to 2018. Padilha et al. found that the increase in accumulated deforestation decreased malaria cases in Rondônia, contrasting with the random effect associated with deforestation that did not find a change after 2015, implying that deforestation might have maintained a similar impact from previous years [30]. Porto Velho, the capital of Rondônia, has presented a high rate of human mobility and malaria transmission in periurban areas linking with the positive random effect of travelling activities [31].

The current results showed that Roraima experienced a rebound of malaria cases in eight municipalities (seven for *P. vivax* and two for *P. falciparum*). However, the accumulated deforestation random effect only had an increase over *P. vivax* after 2015, suggesting that economic variables and imported cases maintained the same effect as previous years had. Previous works [32] found the socioeconomic situation in Venezuela to have significant effects on the malaria rebound in Roraima. However, the current results did not show a positive effect associated with imported cases, placing more importance on autochthonous cases or possibly not capturing the migration effect. Migration might also account for an increase in deforestation effect that might explain the positive random effect after 2015 [33].

The results did not show a clear driver of malaria increase in Acre, however previous studies indicated fish farming in the last decade had provided environmental advantages to *Anopheles* mosquitoes construction in Acre. Priority should be given to health attention, and Acre also has experienced an increase in economic activities in periphery zones boosting malaria transmission [6, 8, 34]. A previous study found a positive relation between deforestation and malaria incidence at this state in contrast to the non-positive effect with this variable after 2015, suggesting a similar deforestation effect from previous years [30]. Nevertheless, accumulated de-forestation has exhibited a non-linear effect due to the dynamics of environmental conditions in the Amazon basin [35]. In general, current results showed the divergence in possible drivers of malaria rebound because epidemiology of malaria across regions in the Amazon basin is heterogeneous, as Canelas et al. had found [9].

The results also did not indicate a clear driver of malaria in Amapá, despite the unexpected increase of *P. vivax* cases in 2018, suggesting a similar effect of the study variables from previous years. Previous studies have evidenced the fragility of the malaria programme in this state due to the human mobility in the French Guyana border area and the high risk of malaria transmission that the indigenous population had taken [36–38]. Nevertheless, Fig. 2 evidenced the unexpected increase in cases in the distant municipalities from the border area, implying an internal cause of the increase in malaria cases in this state. The malaria rebound in Pará might explain the increase in malaria cases in Amapá by the proximity with Marajó region, the main cluster of malaria rebound in Pará.

The Brazilian-Amazon basin has a multifactorial set of environmental, social, and economic conditions related to the unexpected increase in malaria incidence. First, human activities in the Amazon region have induced deforestation that drives malaria transmission, even though a recent study also showed negative feedback where malaria burden reduces forest clearing [39]. Malaria incidence and deforestation have a relation because human development and environmental conditions promote a dynamic where deforestation can increase malaria burden [40]. The current results showed that deforestation had a positive effect on *P. vivax* incidence in Roraima from 2015 to 2018, despite not allowing to refute this effect in the unexpected increase of cases in other areas of the Amazon basin because the unimodal relationship between deforestation and malaria burden might mask a positive or negative effect of this variable [35]. In addition, the inferences between disease incidence and environmental variables can generate disturbances in the conclusions due to the analysis assumptions [41].

State-based analysis for inferring variable relations has a set of limitations. The first limitation is the variation between municipalities because mixed random effects between municipalities can underestimate the impact of some variables. Secondly, a few municipalities in the Amazon basin accounted for most of malaria cases, and the inclusion of a state analysis can also drive an underestimation of variable effects [22]. However, the current study found a set of plausible causes in malaria rebound for some states from 2015 to 2018 and also found a spatio-temporal pattern of malaria cases at this period.

## Conclusion

The Brazilian Amazon basin has experienced an unexpected increase in malaria cases, mainly in *P. vivax* cases, in some regions of the states of Amazonas, Acre, Pará, Amapá, Roraima, and Rondônia from 2015 to 2018 and agricultural activities, outdoor activities, travelling activities, and accumulated deforestation appear linked to this rebound of cases in particular regions with different impact. This reveals the heterogeneity of the Amazon basin, demonstrating the necessity of focusing the malaria control programme on the particular social, economic and environmental conditions.

## Supplementary Information

**Additional file 1: Figure S1. **Study area: consists in nine states located in Northwest region surrounding by Colombia, Venezuela, Peru, Bolivia, Suriname and French Guiana.

**Additional file 2: Table S1. **Choice criteria between model 1 and model 2 in all States.

## Data Availability

Epidemiological data on malaria cases are available upon request to the Brazilian Ministry of Health. The integrated dataset from Baroni et al*.* [10] also contains the SIVEP data on malaria cases. All scripts for the statistical analysis are available from the URL: https://github.com/Mario-Canon-Ayala/On-multifactorial-drivers-for-malaria-rebound-in-Brazil.

## References

[1] PAHO/WHO (2018). Epidemiological alert: increase of malaria in the Americas.

[2] WHO (2018). World Malaria report 2018.

[3] Carter KH, Singh P, Mujica OJ, Escalada RP, Ade MP, Castellanos LG (2015). Malaria in the Americas trends from 1959 to 2011. Am J Trop Med Hyg.

[4] de Pina-Costa A, Brasil P, Di Santi SM, de Arujo PM, Suaréz-Mutis MC, Silva Santelli AN (2014). Malaria in Brazil: what happens outside the Amazonian endemic region. Mem Inst Oswaldo Cruz.

[5] Souza PF, Xavier DR, Suarez Mutis MC, da Mota JC, Peiter PC, de Matos VP (2019). Spatial spread of malaria and economic frontier expansion in the Brazilian Amazon. PLoS ONE.

[6] dos Reis IC, Codeco CT, Degener CM, Keppeler EC, Muniz MM, de Oliveira FG (2015). Contribution of fish farming ponds to the production of immature *Anopheles* spp. in a malaria-endemic Amazonian town. Malar J.

[7] Cohen JM, Smith DL, Cotter C, Ward A, Yamey G, Sabot OJ (2012). Malaria resurgence: a systematic review and assessment of its causes. Malar J.

[8] Corder RM, Paula GA, Pincelli A, Ferreira MU (2019). Statistical modeling of surveillance data to identify correlates of urban malaria risk: a population-based study in the Amazon Basin. PLoS ONE.

[9] Canelas T, Castillo-Salgado C, Ribeiro H (2018). Analyzing the local epidemiological profile of malaria transmission in the Brazilian Amazon between 2010 and 2015. PLoS Curr.

[10] Baroni L, Pedroso M, Barcellos C, Salles R, Salles S, Paixão B (2020). An integrated dataset of malaria notifications in the Legal Amazon. BMC Res Notes.

[11] Brazilian Institute of Geography and Statistic (IBGE). Population. https://www.ibge.gov.br/estatisticas/sociais/populacao.html. Accessed Mar 2019.

[12] Brazilian Institute of Geography and Statistic (IBGE). Cartography and maps downloads. https://www.ibge.gov.br/geociencias/downloads-geociencias.html. Accessed Mar 2019.

[13] PRODES project of National Institute of Spatial Research. Deforestation per Brazilian municipalities. http://www.dpi.inpe.br/prodesdigital/prodesmunicipal.php. Accessed Sept 2019.

[14] Lindgren F, Rue H (2008). On the second-order random walk model for irregular locations. Scand Stat Theory Appl.

[15] Besag J, York J, Mollie A (1991). Bayesian image restoration, with two applications in spatial statistics. Ann Inst Stat Math.

[16] Blangiardo M, Cameletti M, Baio G, Rue H (2013). Spatial and spatio-temporal models with R-INLA. Spat Spatiotemporal Epidemiol.

[17] Rue H, Martino S, Chopin N (2009). Approximate Bayesian inference for latent Gaussian models by using integrated nested Laplace approximations. J R Stat Soc Ser B.

[18] Spiegelhalter DJ, Best NG, Carlin BP, van der Linde A (2002). Bayesian measures of model complexity and fit. J R Stat Soc Ser B.

[19] Sarkar D (2008). Lattice: multivariate data visualization with R.

[20] Wickham H (2016). ggplot2: elegant graphics for data analysis.

[21] Carlos BC (2019). Comprehensive analysis of malaria transmission in Brazil. Pathog Glob Health.

[22] Lana R, Nekkab N, Siqueira AM, Peterka C, Marchesini P, Lacerda M (2021). The top 1%: quantifying the unequal distribution of malaria in Brazil. Malar J.

[23] Lima ID, Lapouble OM, Duarte EC (2017). Time trends and changes in the distribution of malaria cases in the Brazilian Amazon Region. Mem Inst Oswaldo Cruz.

[24] Sousa JR, Santos ACF, Almeida W, Albarado Kaio VP, Magno LD, Rocha JA (2015). Situação da malária na região do Baixo Amazonas, estado do Pará, Brasil, de 2009 a 2013: um enfoque epidemiológico. Rev Pan Amaz Saúde.

[25] Pereira ALRR, Miranda CDSC, Guedes JA, Oliveira RAC, Campos PSDS, Palácios VRDCM (2021). The socio-environmental production of malaria in three municipalities in the Carajás region, Pará, Brazil. Rev Saude Publica.

[26] Ueno TMRL, Lima LNGC, Sardinha DM, Rodrigues YC, Souza HUS, Teixeira PR (2021). Socio-epidemiological features and spatial distribution of malaria in an area under mining activity in the Brazilian Amazon Region. Int J Environ Res Public Health.

[27] Melo JO, Padilha MAO, Barbosa RTA, Alonso WJ, Vittor AY, Laporta GZ (2020). Evaluation of the malaria elimination policy in Brazil: a systematic review and epidemiological analysis study. Trop Biomed.

[28] Meireles BM, de Souza Sampaio V, Monteiro WM, Goncalves MJF (2020). Factors associated with malaria in indigenous populations: a retrospective study from 2007 to 2016. PLoS ONE.

[29] Ferreira MU, Castro MC (2016). Challenges for malaria elimination in Brazil. Malar J.

[30] de Oliveira Padilha MA, de Oliveira MJ, Romano G (2019). Comparison of malaria incidence rates and socioeconomic-environmental factors between the states of Acre and Rondônia: a spatio-temporal modelling study. Malar J.

[31] Angelo JR, Katsuragawa TH, Sabroza PC, de Carvalho LA, Silva LH, Nobre CA (2017). The role of spatial mobility in malaria transmission in the Brazilian Amazon: the case of Porto Velho municipality, Rondônia, Brazil (2010–2012). PLoS ONE.

[32] Recht J, Siqueira AM, Monteiro WM, Herrera SM, Herrera S, Lacerda MVG (2017). Malaria in Brazil, Colombia, Perú and Venezuela: current challenges in malaria control and elimination. Malar J.

[33] PAHO (2016). Plan of action for malaria elimination 2016–2020 (CD55/13). 55th directing council, 68th session of the PAHO regional committee.

[34] Alves MR, CodeçoPeiter CTPC, Souza-Santos R (2019). Malaria and fish farming in the Brazilian Amazon Region: a strengths, weaknesses, opportunities, and threats analysis. Rev Soc Bras Med Trop.

[35] Laporta GZ (2019). Amazonian rainforest loss and declining malaria burden in Brazil. Lancet Planet Health.

[36] da Cruz FV, Peiter PC, Carvajal-Cortés JJ, Dos Santos PR, Mendonça Gomes MDS, Suárez-Mutis MC (2019). Complex malaria epidemiology in an international border area between Brazil and French Guiana: challenges for elimination. Trop Med Health.

[37] Mosnier E, Dusfour I, Lacour G, Saldanha R, Guidez A, Gomes MS (2020). Resurgence risk for malaria, and the characterization of a recent outbreak in an Amazonian border area between French Guiana and Brazil. BMC Infect Dis.

[38] Mendes AM, Lima MDS, Maciel AGP, Menezes RAO, Eugênio NCC (2020). Malaria among indigenous peoples on the Brazil-French Guiana border, 2007–2016: a descriptive study. Epidemiol Serv Saude.

[39] MacDonald AJ, Mordecai EA (2019). Amazon deforestation drives malaria transmission, and malaria burden reduces forest clearing. Proc Natl Acad Sci USA.

[40] Chaves LSM, Conn JE, Lopez RVM, Sallum MAM (2018). Abundance of impacted forest patches less than 5 km^2^ is a key driver of the incidence of malaria in Amazonian Brazil. Sci Rep.

[41] Valle D, Laporta GZ (2021). A cautionary tale regarding the use of causal inference to study how environmental change influences tropical diseases. Am J Trop Med Hyg.

