# Supplementary material for: On multifactorial drivers for malaria rebound in Brazil: a spatio-temporal analysis
Source: Malar J. 2022 Feb 17;21:52. doi: 10.1186/s12936-021-04037-x (PMC8851784; doi:10.1186/s12936-021-04037-x)
Supplement: Supplementary file 1 — Additional file 1: Figure S1. Study area: consists in nine states located in Northwest region surrounding by Colombia, Venezuela, Peru, Bolivia, Suriname and French Guiana. [file 12936_2021_4037_MOESM1_ESM.pdf]

On multifactorial drivers for malaria rebound: a spatio-temporal analysis

Mario J.C. Ayala[1], **Leonardo S Bastos**[1], **and Daniel A.M. Villela**[1,*]

[1]Fundação Oswaldo Cruz (Fiocruz), Programa de Computação Científica, Rio de Janeiro, 21.040-900, Brazil

Additional Files

Additional file 1 — Study area

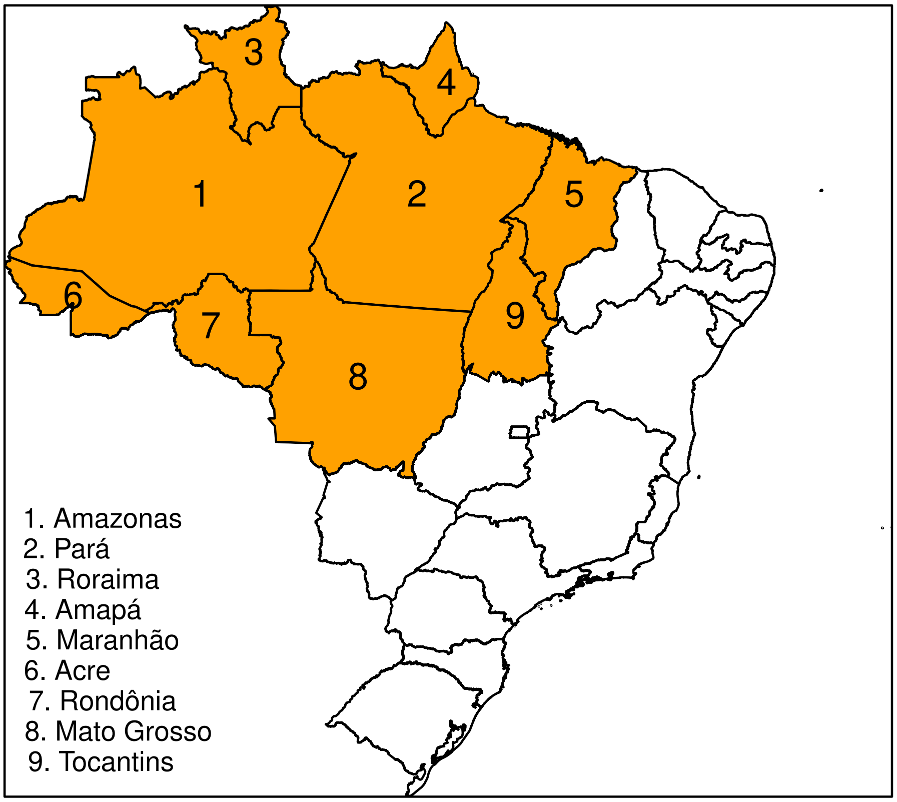

Figure 1. Study area: consists in nine states located in Northwest region surrounding by Colombia, Venezuela, Peru, Bolivia, Suriname and French Guiana.
