# Supplementary material for: On multifactorial drivers for malaria rebound in Brazil: a spatio-temporal analysis
Source: Malar J. 2022 Feb 17;21:52. doi: 10.1186/s12936-021-04037-x (PMC8851784; doi:10.1186/s12936-021-04037-x)
Supplement: Supplementary file 2 — Additional file 2: Table S1. Choice criteria between model 1 and model 2 in all States. [file 12936_2021_4037_MOESM2_ESM.pdf]

Additional file 2 — Model choice

Table 1. Choice criteria between model 1 and model 2 in all States.

State	Model	DIC *P. vivax*	DIC *P. falciparum*
AM	Model 1	755556.9	219725.7
AM	Model 2	733638.3	215179.9
PA	Model 1	691275.9	174913.5
PA	Model 2	670231.9	168723.3
AC	Model 1	142410	74157.6
AC	Model 2	127973.5	64414.6
AP	Model 1	63918.9	34309.1
AP	Model 2	59017.5	31485.6
RR	Model 1	79208.7	19119.7
RR	Model 2	71863.2	18198.3
RO	Model 1	140897.5	54656.7
RO	Model 2	132188.6	50360.7
MA	Model 1	91185.4	31755
MA	Model 2	88674.5	30904.9
TO	Model	7342.4	2857.4
TO	Model 2	7202.2	2806.4
MT	Model 1	50597.4	16439.9
MT	Model 2	49091.3	15406.9
